# Soil erosion and land-use management influence bioavailable selenium in a highland agricultural landscape in Kenya

**DOI:** 10.1007/s10653-026-03557-2

**Published:** 2026-10-24

**Authors:** Job Isaboke, Olivier S. Humphrey, Odipo Osano, Ruth Njoroge, Michael J. Watts

**Affiliations:** 1 Department of Environmental Science, School of Environmental Sciences and Natural Resources Management, University of Eldoret, Eldoret, Kenya; 2 Inorganic Geochemistry, Centre for Environmental Geochemistry, British Geological Survey, Nottingham, NG12 5GG UK; 3 Department of Soil Science, School of Agriculture and Biotechnology, University of Eldoret, Eldoret, Kenya

**Keywords:** Selenium, Soil, Bioavailability, Kenya

## Abstract

Selenium (Se) is an essential micronutrient whose availability in agricultural systems is influenced by geochemical, pedological, and anthropogenic factors. The study aimed to investigate the effects of soil erosion, land-use, and management practices on the bioavailability and redistribution of selenium within a soil–plant continuum in a highland agricultural landscape in Nandi County, Kenya. A stratified sampling approach was applied across three plots representing distinct land-use management practices and varying soil erosion rates. Selenium concentrations were measured both in soils and crops (maize, beans, and pumpkin) collected from the experimental sites. Total and bioavailable Se concentrations in soils were evaluated using a mixed acid digestion (HF, HNO_3_, and H_2_O_2_) and an ammonium chloride (NH_4_Cl) extraction, respectively, followed by ICP-MS analysis. Structural equation modelling and regression analyses revealed that soil pH (β = − 0.41, *p* < 0.001), organic matter (β = 0.29, *p* < 0.01), and total Se (β = 0.30, *p* < 0.001) significantly influenced bioavailable Se, although plant uptake showed weak correlation. Temporal comparisons showed a significant increase in bioavailable Se in Plot 3 from 6.74 µg kg^−1^ (2021) to 10.03 µg kg^−1^ (2023), representing a 42% rise. In contrast, Plot 1 experienced a significant decline (*p* < 0.05) from 14.72 µg kg^−1^ to 9.56 µg kg^−1^ over the same period, linked to an increase in erosion rate by 0.58 t ha^−1^ yr^−1^. Maize showed significantly higher Se accumulation in Plot 1 (187 µg kg^−1^) compared to Plot 3 (87 µg kg^−1^) (*p* < 0.05), while beans and pumpkin showed no significant differences across plots (*p* > 0.05). These findings suggest that appropriate land management and erosion mitigation may help maintain soil Se bioavailability, while targeted agronomic strategies could be considered to enhance crop Se concentrations. However, the relatively low Se concentrations observed in several crops suggest a potential concern regarding Se adequacy, and assessment of dietary intake and human biomarkers would be required to establish nutritional risk.

**Graphical abstract:**

**Figure:**
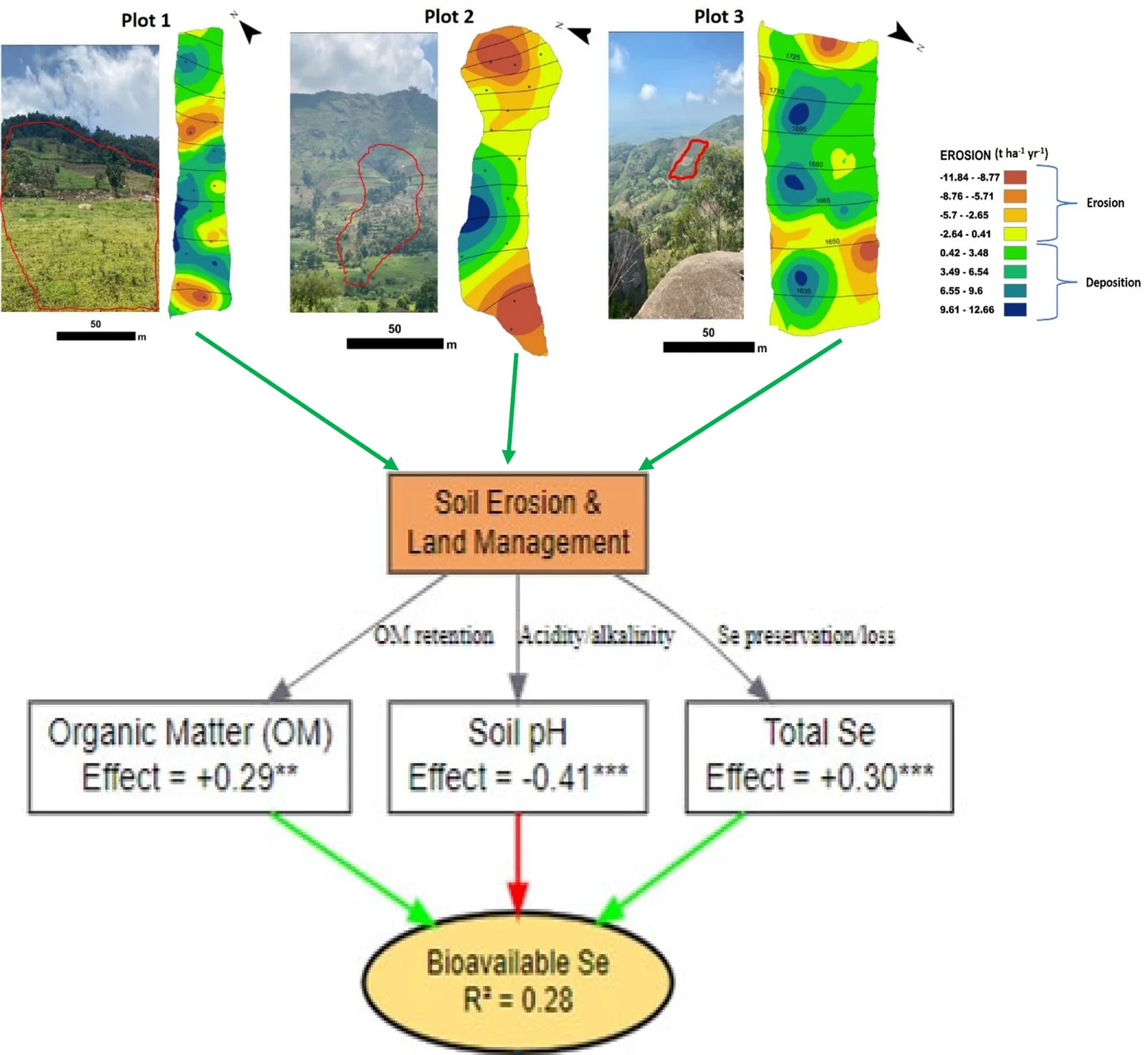

**Supplementary Information:**

The online version contains supplementary material available at https://doi.org/10.1007/s10653-026-03557-2.

## Highlights

- Soil erosion and land use influence soil selenium bioavailability.
- Despite soil selenium concentrations, crops show poor uptake efficiency.
- Integrated soil management and biofortification may inform local biofortification strategies.

**Supplementary Information:**

The online version contains supplementary material available at https://doi.org/10.1007/s10653-026-03557-2.

## Introduction

Selenium (Se) is a vital but relatively scarce trace element whose concentration in soils varies markedly across different regions due to natural geochemical and pedological processes (Fordyce, 2013; Saha et al., 2016; Eze et al., 2025). These spatial disparities are largely influenced by factors such as parent rock composition, soil pH, redox conditions, organic matter content (OM), soil erosion rates, and climatic variables (Long et al., 2022; Xie et al., 2023). Selenium is an essential micronutrient for both humans and animals, playing critical roles in numerous physiological and biochemical processes. In humans, at least 25 selenoproteins have been identified, many of which function as components of antioxidant defence systems, thyroid hormone metabolism, and immune response modulation (Hariharan & Dharmaraj, 2020; Prasad et al., 2025). These selenoproteins include glutathione peroxidases (GPx), thioredoxin reductases (TrxR), and selenoprotein P, which are pivotal in mitigating oxidative stress, reducing inflammation, and potentially offering protection against viral infections and carcinogenesis (Hariharan & Dharmaraj, 2020; Shahidin et al., 2025).

In environmental matrices, selenium exists in several chemical forms that influence its bioavailability and mobility. The most common inorganic forms in soils are selenite (SeO_3_^2−^) and selenate (SeO_4_^2−^), typically present as sodium salts such as sodium selenite (Na_2_SeO_3_) and sodium selenate (Na_2_SeO_4_) (McNeal & Balistrieri, 1989). Organic forms, including selenocysteine and selenomethionine, are found in biological tissues and contribute to Se cycling through the soil–plant continuum. Selenium can also exist in solid elemental forms under certain reducing conditions, often rendering it less bioavailable (Antonyak et al., 2018; Mehdi et al., 2013).

A distinctive feature of Se is its narrow physiological range between deficiency and toxicity. While moderate Se intake supports essential biological functions, deviations on either side can have detrimental health consequences (Fairweather-Tait et al., 2010). Deficiency is associated with disorders such as Keshan disease and impaired immune function, while excessive intake can lead to selenosis, characterised by symptoms such as gastrointestinal distress, hair loss, and neurological disturbances (Bai et al., 2025; Shreenath et al., 2023). Therefore, maintaining an optimal selenium status is critical for health, particularly in regions where soil Se concentrations are naturally low, are highly variable or have poor transfer rates to crops (Ekumah et al., 2021).

Selenium (Se) is generally present in low concentrations in most rocks. Sedimentary rocks typically contain more Se than igneous rocks, though levels in limestones and sandstones rarely exceed 0.1 mg kg^−1^ (Presser, 1999; Ralston et al., 2008). Higher concentrations are often found in the clay-rich fractions of sediments, with shales averaging ~0.06 mg kg^−1^. Notably, elevated Se levels, up to 300 mg kg^−1^, can occur in phosphatic rocks (Fordyce, 2013). Organic-rich materials such as coal and black shales may be significantly enriched in Se, with typical values ranging from 1–20 mg kg^−1^ and occasionally exceeding 600 mg kg^−1^. Selenium is commonly associated with sulphide minerals, while elemental selenium (Se) is rare (Malisa, 2001). These variations reflect the geochemical diversity of rock types and their influence on Se distribution in the environment (Michalke, 2018).

Selenium deficiency affects over 25% of Africa’s population, with Southern Africa facing particularly high dietary inadequacy risks (Farooq et al., 2023; Hurst et al., 2013). This geospatial variability is largely driven by soil properties, especially pH, organic matter, and parent material. In Malawi, for example, populations in regions with calcareous soils exhibit higher selenium status due to enhanced selenium bioavailability at higher pH levels compared to areas with acidic soils (Chilala et al., 2024). In addition, in certain seleniferous regions, concentrations as high as 1200 mg kg⁻^1^ have been documented. The dominant inorganic forms in soils are selenite (Se^4+^) and selenate (Se^6+^). Selenite binds strongly to soil particles, particularly under acidic conditions and in the presence of iron oxides and organic matter, thereby reducing its bioavailability. Selenate, in contrast, remains more soluble and mobile, and is readily taken up by plants in neutral to alkaline soils. Elemental selenium (Se) and selenides (Se^2−^) typically form under reducing, acidic, and organic-rich conditions and are not readily accessible to biota. Understanding Se bioavailability is crucial for evaluating potential health risks, as total Se content alone may not reflect its nutritional or toxicological significance. Among various assessment methods, water-extractable Se, commonly below 0.1 mg kg^−1^ in most soils, is widely used as an indicator of available Se (Perrone et al., 2015). The mobility and distribution of selenium (Se) in agricultural soils are significantly influenced by soil erosion, texture, and land management practices. Soil erosion, particularly water and wind erosion, can lead to the selective removal of fine, Se-rich particles such as clay and organic matter, resulting in a net depletion of Se in the topsoil and potential enrichment in downstream sediments (Lopes et al., 2017). Soil texture plays a crucial role in regulating Se retention and leaching; finer-textured soils (e.g., clays and silty loams) tend to retain more Se through adsorption processes, especially in the form of selenite, which binds to iron and aluminium oxides. In contrast, coarse-textured soils have lower adsorption capacity, increasing the potential for selenate leaching under well-aerated conditions (Imran et al., 2023). Land management practices such as tillage, irrigation, and the application of fertilisers and organic amendments also affect Se cycling. For instance, reduced tillage can minimise erosion and enhance organic matter retention, thereby improving the presence of Se in the upper soil layers. Similarly, manure and compost inputs may increase organic Se fractions and influence microbial activity that drives Se speciation and transformation (Zhou et al., 2023). Therefore, the interplay between erosion, soil physical properties, and management strategies governs the spatial and temporal dynamics of selenium in agricultural environments.

While selenium is not considered essential for plant growth, it is nonetheless taken up and incorporated into plant tissues, with concentrations generally reflecting environmental availability (Grabowski et al., 2024; Hurst et al., 2013; Mutonhodza et al., 2022). A key factor influencing the risk of Se-related health issues in animals and humans is the broad variability among plant species in their ability to accumulate Se. Early work by Rosenfeld and Beath (1945) classified plants based on Se uptake when grown in seleniferous soils. Hyperaccumulator species can contain over 1000 mg kg^−1^ of Se, whereas non-accumulators such as most cereals and forage grasses typically have concentrations below 50 mg kg^−1^. Some species within genera such as Astragalus, Haplopappus, and Stanleya are known Se accumulators in semi-arid, seleniferous regions, though not all species within these genera exhibit high uptake capacities (Statwick et al., 2016). Micronutrient availability, particularly that of selenium (Se), plays a critical role in sustaining animal health and is obtained from the soil through plants. However, its distribution and bioavailability across the soil–plant–animal continuum are increasingly disrupted by anthropogenic and natural factors. Soil erosion in intensively managed agricultural systems selectively removes fine, nutrient-rich particles, leading to uneven redistribution of micronutrients and reduced availability in the root zone. This process can significantly impact plant uptake and, subsequently, animal nutrition through forage and crop quality (Somagattu et al., 2024). Despite the recognition of these drivers, a limited understanding remains of how erosion and land management interact with soil particle size distribution to influence the movement and transfer of micronutrients through the soil–plant continuum.

Although previous studies have examined selenium distribution in soils and its availability to plants, the relationships between soil particle-size fractions, soil physicochemical properties, selenium bioavailability, and crop selenium accumulation remain insufficiently understood in erosion-affected agricultural landscapes. The extent to which variations in soil organic matter, pH, and total selenium influence bioavailable selenium, and whether these relationships translate into selenium accumulation in different crops, requires investigation. Understanding these relationships is important because total soil selenium concentrations alone may not adequately reflect its availability for plant uptake. This study therefore investigates selenium distribution and bioavailability across soil particle-size fractions and evaluates the relationships between soil properties, bioavailable selenium, and selenium concentrations in maize, beans, and pumpkin in the study area.

This study aimed to address this gap in knowledge by assessing the effects of soil erosion, particle size distribution, and land management practices on the availability and redistribution of selenium in agricultural landscapes, using the following objectives: (a) to assess the impact of soil erosion and land management on the redistribution of selenium across different soil particle size fractions, (b) to assess the bioavailability of selenium for plant and how it is affected by soil erosion and land use management.

## Materials and methods

### Study area

**Fig. 1:**
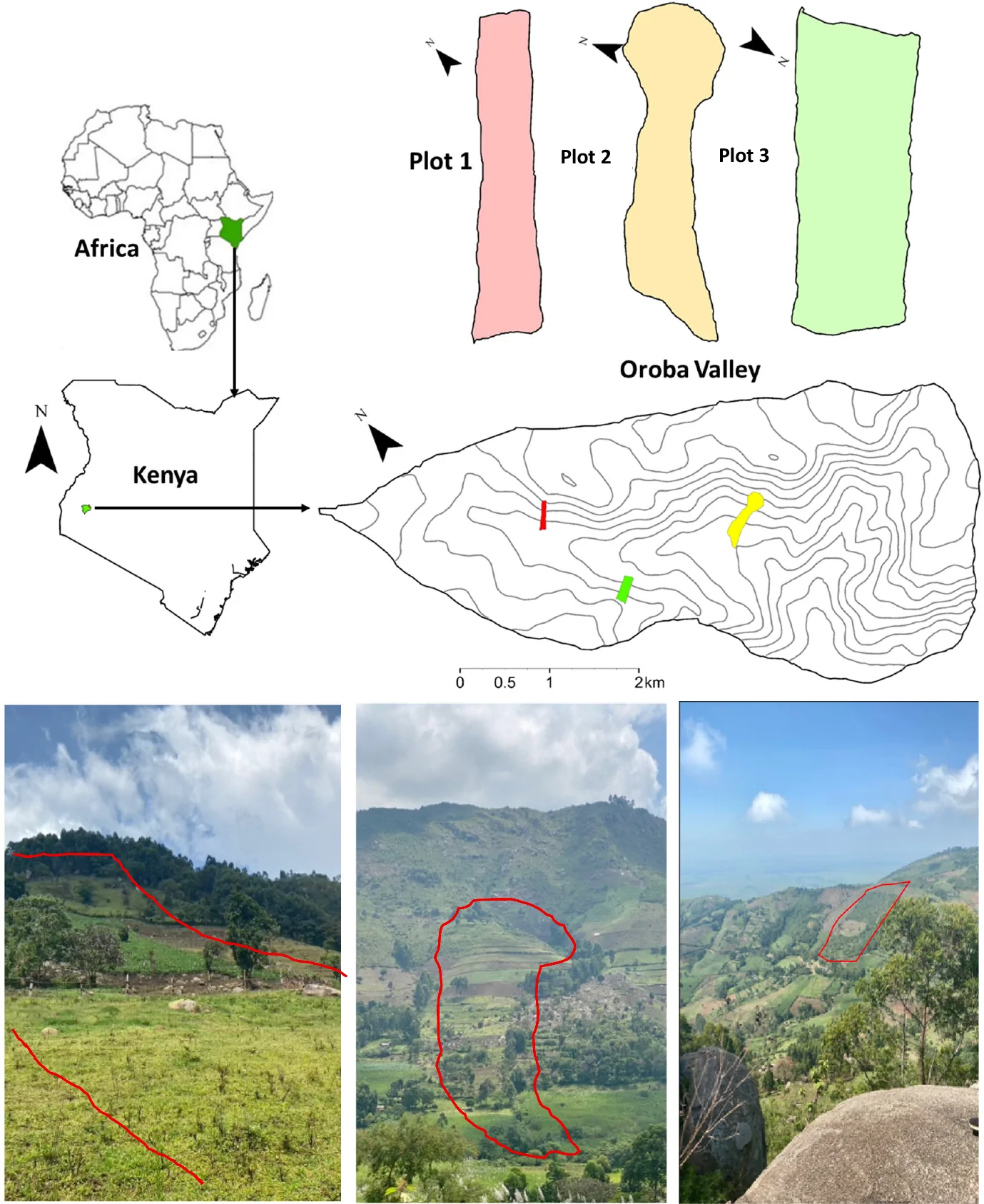
Map of the study area in Oroba Valley, Nandi County, Kenya, showing plots: Plot 1, Plot 2, and Plot 3 along the Oroba River, which drains into Lake Victoria. Elevation ranges from 1900 to 1300 m above sea level

The study was conducted from 2021 to 2023 on three agricultural plots situated along the slopes of the Oroba Valley in Nandi Hills, within Kenya’s Great Rift Valley region. The study sites were located around the following GPS points: Plot 1 (0.007735°N, 34.987602°E), Plot 2 (0.004924°S, 35.003758°E), and Plot 3 (0.003332°S, 34.989053°E) (Fig. 1). The Oroba River runs through the valley and ultimately flows into the Winam Gulf, Lake Victoria. This area lies within the upper highland agroecological zone, characterised by a bimodal rainfall pattern with annual precipitation ranging between 500 and 1200 mm (Kipkulei et al., 2024). As reported by Humphrey et al. (2022), the region is classified as high-risk for erosion due to significant land disturbance from agricultural activities.

The three study plots were selected based on differences in cultivation history, farming practices, and erosion-control strategies. Plots 1 and 2 have been continuously cultivated since the 1940 s, representing over 80 years of land use. In contrast, Plot 3 was recently converted from shrubland to farmland in 2023. This contrast allowed for assessment of how cultivation duration influences soil dynamics (Montgomery, 2007). Plot 1 employed traditional, manual, and conventional methods with increasing intensity over time. Plot 2 employed conventional tillage with oxen and terracing to manage erosion, whereas Plot 3, under maize cultivation, is transitioning from natural vegetation and is partially tilled using conventional methods with handheld hoes.

### Sampling design

Soil sampling was conducted using a stratified sampling strategy guided by topographic variation within each of the three plots. Each plot was subdivided into five or six elevation-based sections (S1–S5/S6), spanning from the highest elevation at approximately 1900 m (S1) to the lowest at approximately 1300 m (S5/S6), with three random sampling points selected within each section. At each point, a 1 m × 1 m area was marked, and topsoil samples (0–20 cm depth) were collected. Composite samples were formed from three auger sub-samples randomly taken within each marked area. Plots 1 and 2 yielded 15 composite samples each (from five sections), while Plot 3 provided 18 samples (from six sections). All samples were stored in Kraft paper bags, transported to the Biotechnology Laboratory at the University of Eldoret for air-drying and preparation, and subsequently sent to the British Geological Survey (Keyworth, Nottingham, UK) for laboratory analysis.

Plant tissue sampling was done at the same georeferenced locations as the soil sampling points. At each sampling point, a 2 × 2 m plot was demarcated and planted with maize (Z*ea mays*), beans (*Phaseolus vulgari*s), and pumpkin (*Cucurbita pepo*) supplied by farmers. No fertilisers or irrigation were applied within the experimental plots; however, farmers continued their normal planting practices in the surrounding areas, including the use of fertilisers. Four months after planting, aboveground plant biomass was harvested, rinsed with distilled water to remove surface contaminants, and stored in Kraft paper bags. Samples were then transported to the University of Eldoret Biotechnology Laboratory for freeze-drying and later ground into a fine powder. The processed samples were subsequently sent to the Inorganic Geochemistry Facility at the British Geological Survey (Nottingham, UK) for inorganic nutrient analysis.

### Estimation of soil redistribution using fallout plutonium radionuclides

The redistribution of soil was quantified using the fallout radionuclide ^239+240^Pu, which was used as a tracer of long-term soil movement. Measured ^239+240^Pu activities (Bq kg⁻^1^) were converted to inventories (Bq m⁻^2^) using the mass depth of the < 2 mm soil fraction, and cumulative inventories across the sampled soil layers were compared with a reference ^239+240^Pu profile representing the baseline distribution of fallout in the soil. Soil redistribution rates were subsequently estimated using the MODERN (Modelling Deposition and Erosion Rates with Radio-Nuclides) model, which converts differences between measured and reference inventories into the depth and rate of redistributed soil. Where the measured inventory was lower than the reference inventory, the site was interpreted as eroded, whereas inventories greater than the reference indicated deposition. For eroded profiles, deeper soil layers were incorporated to a depth of 60 cm using an exponential decline in fallout radionuclide activity, while for depositional profiles, additional surface layers were used to represent sediment accumulation. The approach assumes that ^239+240^Pu originates predominantly from global fallout, that the reference profile represents the baseline ^239+240^Pu distribution, and that the deposition depth corresponds to the reference plough depth.

The erosion rate was calculated using the MODERN formulation, where *x′* represents the rate of soil redistribution with depth, *xₘ* is the soil mass depth at the sampling location, *d* is the measured depth increment, and *t₁–t₀* represents the elapsed time between sampling and the main ^239+240^Pu fallout period, taken as approximately 1963. Net soil redistribution was calculated from the difference between soil loss and soil gain, with the resulting spatial patterns interpolated using Ordinary Kriging in ArcGIS. For interpretation of the maps, positive values represented deposition and negative values represented net soil loss. The same stratified sampling framework and plot sections were assessed in 2021 and 2023, allowing the modelled redistribution patterns and rates to be compared between the two sampling periods. The 2021–2023 comparison, therefore, represents changes in the spatial distribution and magnitude of modelled soil redistribution associated with changes in land management and vegetation cover. Kriging variance was used to quantify spatial prediction uncertainty, with corresponding uncertainty maps provided in the supplementary material (Dowell et al., 2024a; 2024b) Isaboke et al., 2026).

### Soil organic matter and pH

Soil organic matter (OM) was quantified using the loss-on-ignition (LOI) method, whereby 1 g of soil was oven-dried at 110 °C for 24 h and subsequently ashed at 450 °C in a muffle furnace. OM content was calculated from the mass loss following ignition, according to standard LOI procedures. Soil pH was determined following the US EPA SW-846 Test Method 9045D for calcareous soil pH analysis. Briefly, 5 g of milled soil particle size fractions (SPSFs) was mixed with 12.5 mL of 0.01 M calcium chloride (CaCl_2_), giving a soil-to-solution ratio of 1:2.5. The suspension was stirred, and the pH was measured (Isaboke et al., 2023).

### Soil sample digestion for total selenium

Soil samples were air-dried at 40 °C and ground before analysis of macro- and micronutrient content. For each sample, 0.25 g was digested using a programmable hot block and a mixed acid solution composed of 2.5 mL hydrofluoric acid (HF), 2 mL nitric acid (HNO_3_), and 2.5 mL hydrogen peroxide (H_2_O_2_), following the procedures outlined by (Watts et al., 2020).

### Bioavailable selenium extraction

To determine bioavailable selenium (Se) in soil, 2 g of air-dried and sieved soil was accurately weighed and placed into a 15 mL polypropylene tube. A 1 M ammonium chloride (NH_4_Cl) extractant was prepared by dissolving 67.4 g of NH_4_Cl in 1.25 L of deionised water. Subsequently, 10 mL of the NH_4_Cl solution was added to each soil sample tube. The mixture was gently agitated on a reciprocating shaker at room temperature for 30 min to facilitate extraction. Following shaking, the samples were centrifuged at 3000 rpm for 15 min to separate the soil particles from the solution. The resulting supernatant was carefully decanted and stored in clean plastic containers for Se analysis.

### Plant digestion

Plant samples were ground (KM-1500 grinder), and 0.5 g of the sample was weighed for digestion using a microwave-assisted digester (MARS Xpress, CEM) for trace and macronutrients. For digestion, 10 mL of 65% HNO_3_ was added to each sample, shaken to mix, and left for 30 min. The PTFE container was capped, placed in the microwave, and heated to 100 °C for 15 min. The vessels were cooled, and 1 mL of H_2_O_2_ was added. The solution was then left for 30 min. The solution was heated again at 200 °C for 25 min. After cooling, 30 mL of deionised water was added to the vessel, and then the mixture was poured into a 60 mL Nalgene bottle. The vessel was rinsed with 9 mL of deionised water to prepare a final solution of 50 mL for subsequent ICP-MS analysis of 57 elements (Watts et al., 2020).

### Statistical analyses

To examine the complex interrelationships among soil properties and selenium uptake in crops, Structural Equation Modelling (SEM) was employed using the lavaan package in R (Rosseel, 2025). SEM is a covariance-based method suitable for theory-driven analysis and hypothesis testing, allowing for simultaneous estimation of multiple dependent relationships while accounting for measurement errors. In this study, a confirmatory model was specified to evaluate the direct influence of soil factors, including organic matter (OM), soil pH, and total selenium, on bioavailable selenium, as well as the subsequent effect of bioavailable selenium on selenium concentrations in maize, beans, and pumpkin. Model parameters were estimated using maximum likelihood, and standardised path coefficients were visualised in a publication-quality SEM diagram. The model’s performance was evaluated using goodness-of-fit indices and R^2^ values, providing robust insights into the pathways that influence crop selenium content.

### Quality control

The accuracy and reliability of the elemental analysis conducted in this study were rigorously evaluated using a suite of internationally recognised certified reference materials (CRMs). These included spinach leaves (SRM 1570a, NIST, USA), tomato leaves (SRM 1573a, NIST, USA), basalt rock (BCR-2, United States Geological Survey, USA), as well as two soil standards: SRM 2711a (NIST, USA) and BGS 102 (British Geological Survey, UK). The choice of these reference materials ensured that the analytical procedures were validated across a broad spectrum of matrices, encompassing plant tissues, geological samples, and soils. The average recovery for the certified reference materials was 93.0 ± 7.7% (n = 14) for NIST2711a Montana Soil, 96.0 ± 5.5% (n = 27) for USGS BCR-2 Basalt, 98.0 ± 8.1% (n = 15) for BGS102 Ironstone Soil, and 95.3 ± 7.0% (n = 8) NRC MESS-4 Marine Sediment. These results confirm the robustness of the methodology applied in this study.

## Results and discussion

### Distribution of bioavailable selenium in differently managed agricultural lands

**Fig. 2:**
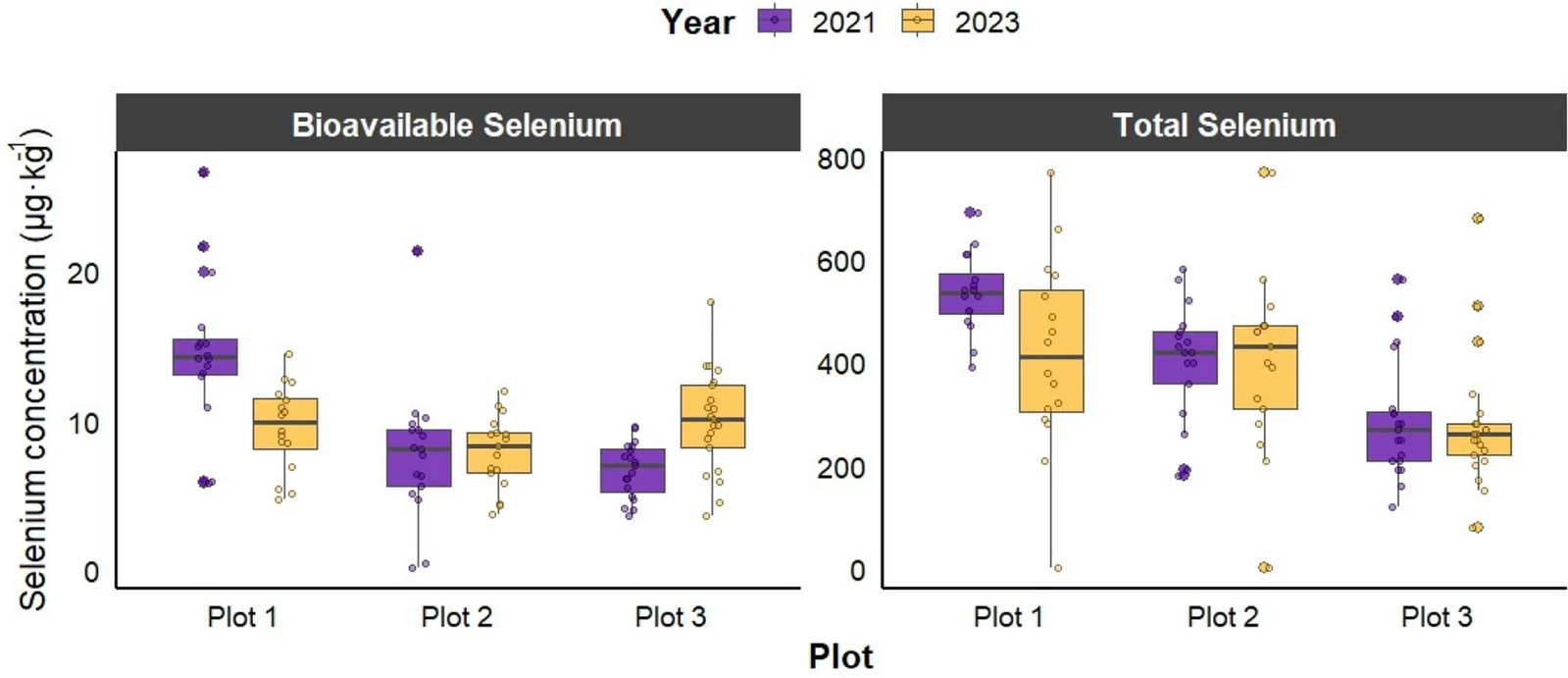
Distribution of total selenium and bioavailable selenium concentrations in soils across study plots for the 2021 and 2023 sampling years

Figure 2 presents the concentration of total and bioavailable selenium (Se) measured across three experimental plots in 2021 and 2023. Total Se concentrations showed considerable variation among plots and between years, reflecting differences in soil properties, environmental conditions, and potential geochemical processes influencing Se accumulation. Bioavailable Se, representing the fraction accessible for plant uptake, was consistently lower than total Se and also varied across plots and years, indicating changes in Se mobility and soil chemical dynamics. Together, these measurements provide a comparative overview of selenium status in the sampled plots and form the basis for evaluating Se trends and availability over time.

The analysis of bioavailable Se across different plots and years reveals important spatial and temporal dynamics that influence soil Se availability. In 2021, Plot 1 exhibited the highest mean concentration of bioavailable Se (14.72 µg kg^−1^). This plot also had a bioavailable Se to total Se proportion of 2.8%. In contrast, Plot 2 had a lower bioavailable Se mean (7.82 µg kg^−1^), reflecting strong spatial heterogeneity in Se distribution, which is driven by differences in soil texture or organic matter content (Isaboke et al., 2026). The low Bioavailable Se/Total Se proportion (2%) in Plot 2 further suggests that Se existed predominantly in less bioavailable forms, possibly bound to soil minerals or organic complexes (Dhillon & Dhillon, 2003). In 2021, Plot 3 had the lowest mean bioavailable selenium (6.74 µg kg^−1^). Interestingly, between 2021 and 2023, a general shift in bioavailable selenium concentrations was observed, particularly in Plot 3, which showed significant (p <0.05) improvement. In 2023, Plot 3 recorded a mean bioavailable selenium of 10.03 µg kg^−1^, 42.0% increase above the bioavailable Se observed in 2021. The increase in both absolute and relative Se bioavailability in Plot 3 may indicate enhanced Se mobilisation in the soil. This pattern could potentially be associated with microbial activity and organic matter decomposition following soil disturbance and turnover resulting from farmer encroachment; Such transformations can convert Se from geochemically stable forms to more plant-accessible species, such as selenate and selenite (Zhu et al., 2009). Plot 1 showed a decline in bioavailable Se concentration from 14.72 µg kg^−1^ in 2021 to 9.56 µg kg^−1^ in 2023, accompanied by a small change in its bioavailable Se to Total Se proportion from 2.8 to 2.0%. This reduction may reflect the depletion of labile Se pools due to continuous uptake by crops and erosion rates (Dhaliwal et al., 2022). Similarly, Plot 2 in 2023 maintained low bioavailable selenium concentrations (7.9 µg kg^−1^) with the lowest relative bioavailability (1.9%), reaffirming its limited Se accessibility for plant uptake. The bioavailable Se/Total Se proportion is critical for estimating the rate of plant uptake. While total Se content provides a baseline, it is the bioavailable fraction that that is linked to the effects of uptake by plants (White & Broadley, 2009). For instance, although Plot 1 in 2021 had the highest bioavailable selenium concentration, Plot 3 in 2023 offered the most efficient Se bioavailability in relative terms, due to more OM and well erosion mitigation strategies that curbed soil movement, which aligns with previous studies that emphasise the complexity of Se cycling in agroecosystems (Joy et al., 2015).

**Fig. 3:**
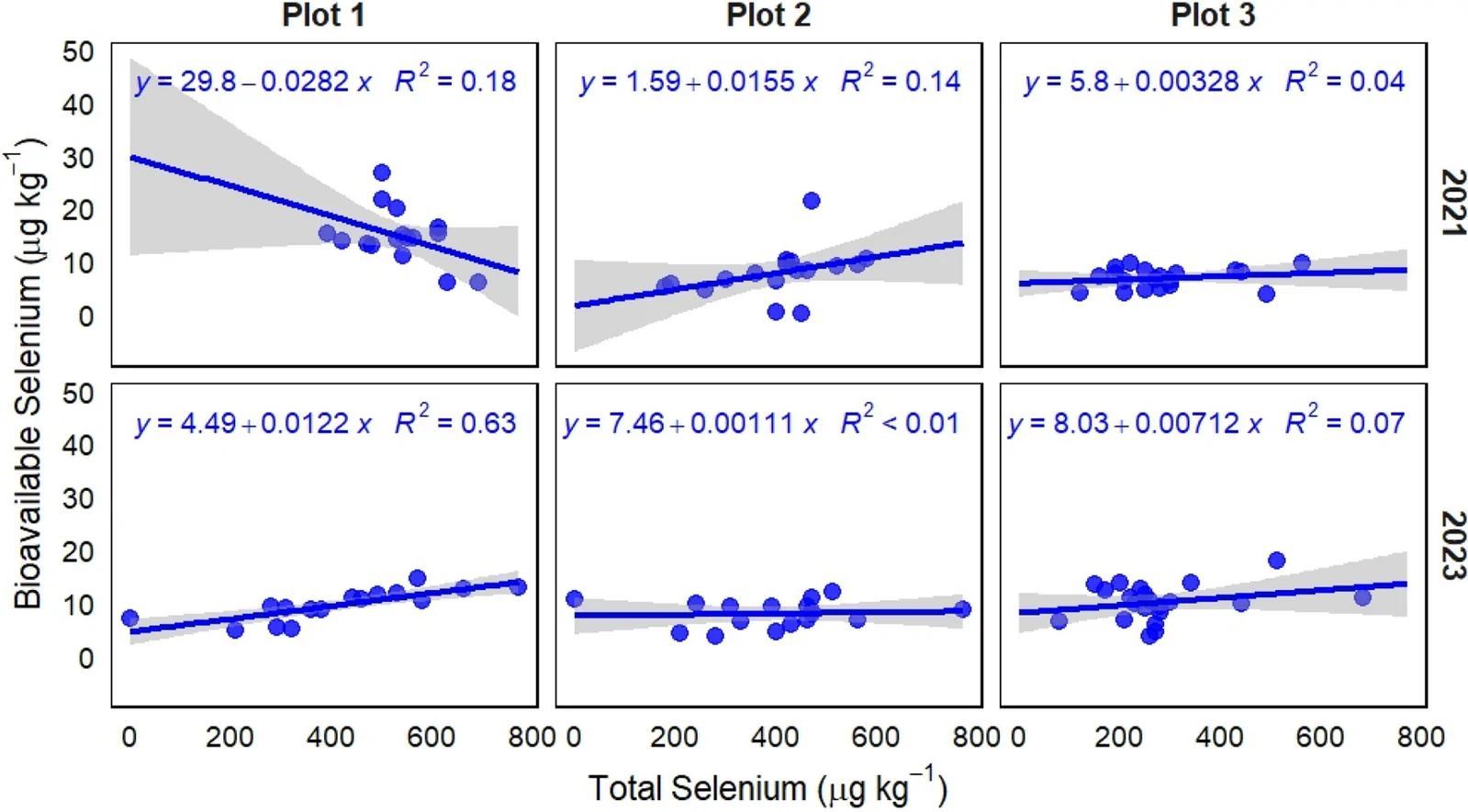
Regression analysis illustrating the relationship between total and bioavailable selenium concentrations across the study plots, differentiated by year of sampling (2021 and 2023)

The observed temporal and spatial changes in bioavailable selenium (Fig. 3) across the study plots appear closely linked to patterns of soil redistribution resulting from erosion and deposition processes, as evaluated through ^239+240^Pu tracer analysis. In 2021, both Plot 1 and Plot 3 exhibited net deposition rates of 0.30 t ha⁻^1^ yr⁻^1^ and 0.72 t ha⁻^1^ yr⁻^1^, (Isaboke et al., 2026)**,** respectively. This depositional environment likely supported the retention of Se, especially in Plot 1, which recorded the highest mean bioavailable Se (15 µg kg^−1^) and a relatively elevated bioavailable Se to Total Se proportion (2.8%). Plot 3, though initially exhibiting lower bioavailable Se (6.7 µg kg^−1^), still maintained a moderately stable Se profile under depositional conditions (proportion = 2.4%).

Plot 2 in 2021 was characterised by significant net soil loss at a rate of −1.66 t ha⁻^1^ yr⁻^1^, which coincided with its high selenium variability and the lowest recorded mean bioavailable selenium concentration (7.8 µg kg^−1^). The bioavailable Se to total selenium proportion was also the lowest at 1.94%, suggesting limited Se availability for plant uptake. These observations suggest that soil erosion plays a role in selectively depleting selenium-rich fine particles and organic matter from the surface soil layer, a process well-documented in degraded agricultural systems (Mu et al., 2022). Erosive forces are known to disproportionately remove topsoil fractions enriched in organic matter and associated micronutrients, such as Se, leading to both reduced bioavailability and increased spatial heterogeneity in elemental distribution (Isaboke et al., 2025; Ngigi et al., 2020). Additionally, the uneven redistribution of soil and Se across the landscape may contribute to localised deficiencies and exacerbate intra-field variability (Isaboke, 2023).

**Fig. 4:**
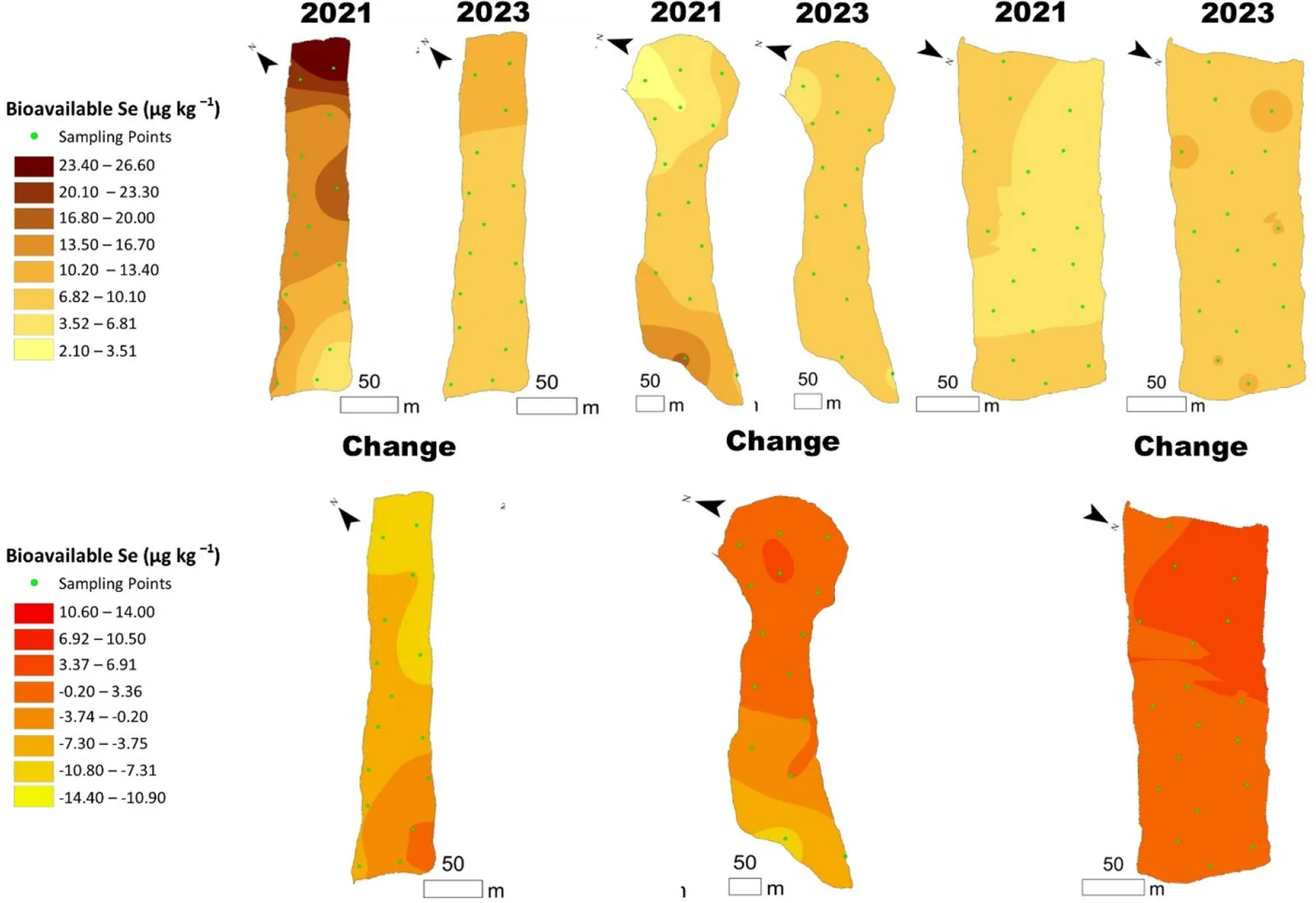
Bioavailable selenium changes over time between 2021 and 2023 on three differently managed agricultural plots in a highly eroded valley

Figure 4: bioavailable selenium changes over time between 2021 and 2023 on three differently managed agricultural plots in a highly eroded valley. Between 2021 and 2023, the erosion regime across all plots shifted markedly toward net soil loss. Plot 1 transitioned from net deposition to net erosion (− 0.58 t ha⁻^1^ yr⁻^1^), accompanied by a reduction in mean bioavailable Se by 5.1 µg kg^−1^ and a decline in Se availability proportion by 0.60%, although the change was not significant (*p* >0.05) continuous loss of top soil through erosion will likely depleted the selenium-enriched soil surface layers (Isaboke et al., 2026). Similarly, Plot 2 continued to exhibit net soil loss (− 0.51 t ha⁻^1^ yr⁻^1^), and although its mean bioavailable Se remained nearly constant, the proportion of bioavailable selenium still declined slightly (0.07%), implying loss of labile selenium forms, but the change was not significant (*p* >0.05). Interestingly, Plot 3 underwent a shift from net deposition in 2021 to net erosion in 2023 (− 0.43 t ha⁻^1^ yr⁻^1^), primarily due to anthropogenic disturbance from farmer encroachment. Despite this change, Plot 3 exhibited an increase in mean bioavailable selenium by 3.3 µg kg^−1^ and a notable rise in bioavailable selenium to Total Se proportion by 1.23%. This counterintuitive trend may reflect enhanced selenium mineralisation, root turnover, or redistribution of selenium from previously enriched depositional zones due to soil disturbance, temporarily boosting selenium availability. When examining cumulative erosion over the two years, the greatest losses of soil occurred in Plot 1 (13.68 ± 3.9 t ha⁻^1^ yr⁻^1^), followed by Plot 2 (6.09 ± 3.2 t ha⁻^1^ yr⁻^1^) and Plot 3 (0.32 ± 3.6 t ha⁻^1^ yr⁻^1^). The Se trends across these plots reflect the erosional intensities: plots with higher cumulative soil loss generally exhibited reduced or stagnated Se availability, except for Plot 3, where soil disturbance may have temporarily increased Se bioavailability (Dinh et al., 2019).

### Impact of soil properties on bioavailable selenium

#### Influence of pH on bioavailable selenium in agricultural soil

**Fig. 5:**
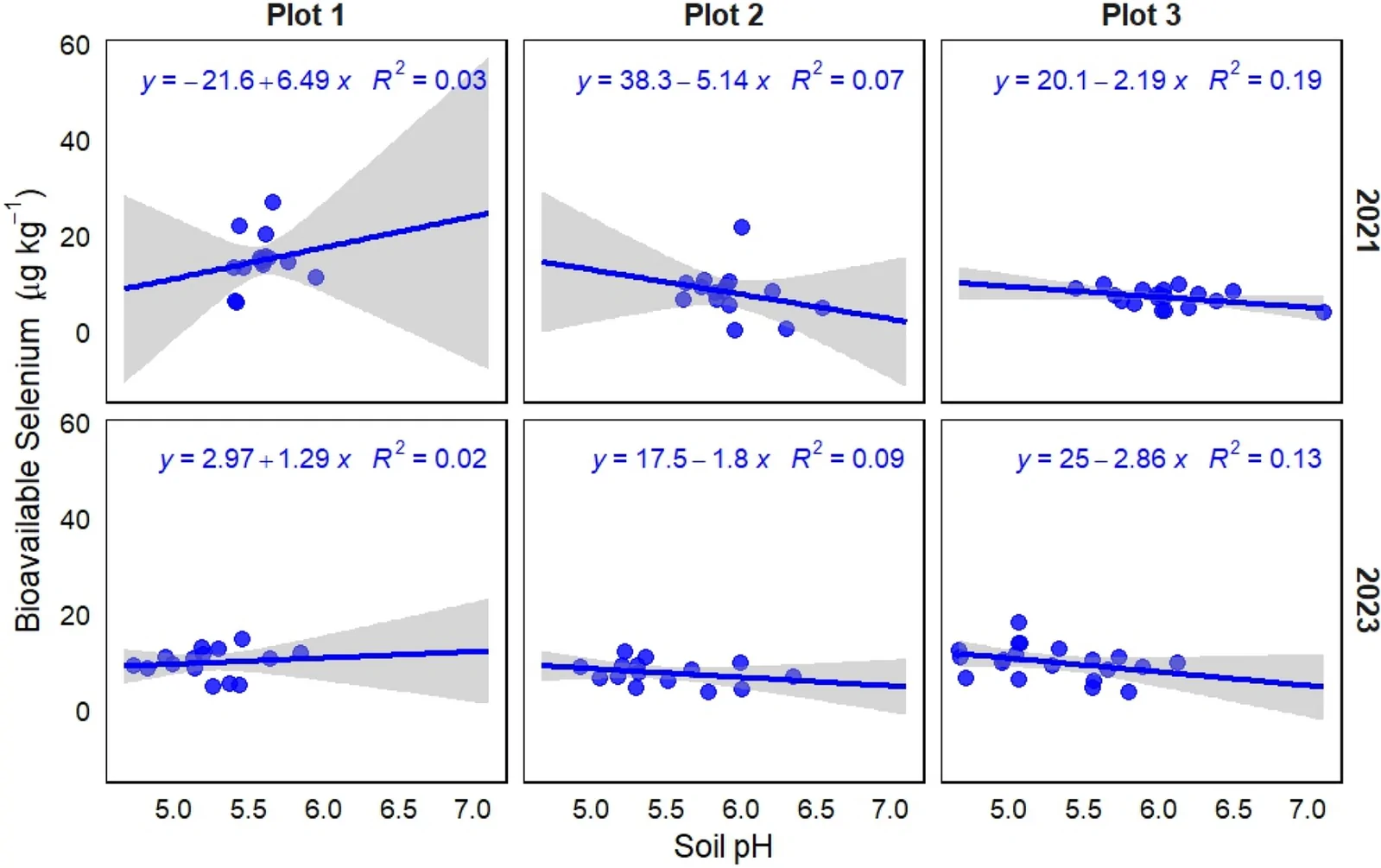
Regression analysis illustrating the relationship between bioavailable and Soil pH across the study plots, stratified by sampling year (2021 and 2023)

Regression analysis revealed no statistically significant relationship between soil pH and bioavailable selenium across all plots and years, as indicated by consistently high p-values (Plot 1 in 2021: *p* > 0.05; Plot 2 in 2021: *p* > 0.05; Plot 3 in 2023: *p* > 0.05). Despite this, negative slope coefficients in Plots 2 and 3 for both years (− 0.00514 in 2021 and − 0.00180 in 2023) suggest an inverse trend between pH and bioavailable selenium (Fig. 5). This aligns with findings by Hurst et al. (2013), who showed that low-pH (acidic) soils in Malawi were associated with lower selenium concentrations in maize and biomarkers of Se status in humans. The mechanism likely involves increased sorption of selenite (Se^4+^) to iron and aluminium oxides under acidic conditions, reducing its mobility and plant availability. Conversely, more neutral or calcareous soils tend to stabilise selenate (Se^6+^), a more bioavailable form, due to weaker adsorption on soil particles. Although the regression results in this study were not statistically significant, the consistent negative slopes indicate a soil-chemistry-driven influence of pH on Se availability, warranting further investigation under experimental conditions. (Fig. 5).

#### Influence of OM on bioavailable selenium in agricultural soil

**Fig. 6:**
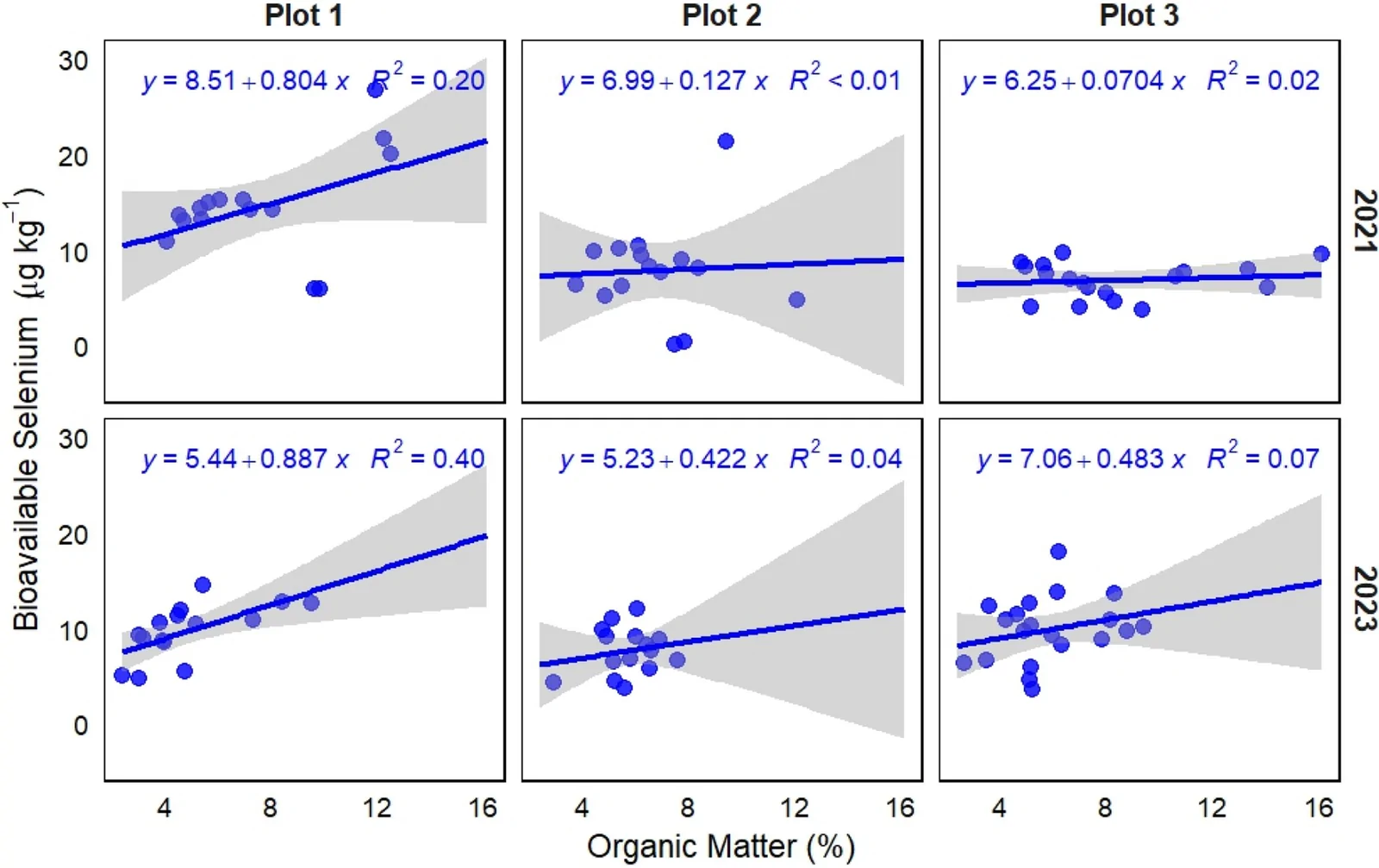
Regression analysis illustrating the relationship between bioavailable and Organic matter concentrations across the study plots, differentiated by year of sampling (2021 and 2023)

The regression analysis between OM and bioavailable selenium across the study plots reveals differences in the strength and significance of their relationships, both between plots and over time. In 2021, the relationships were generally weak and non-significant across all plots. Plot 1 exhibited the strongest relationship for that year, with an R^2^ value of 0.2 (Fig. 6), indicating that approximately 20% of the variability in bioavailable selenium could be explained by OM. In contrast, Plot 2 and Plot 3 showed negligible explanatory power, with R^2^ values of 0.003 and 0.02 (*p* > 0.05), indicating that in these plots, OM had no predictive value for bioavailable selenium (Fig. 6). The weak predictive power of OM for bioavailable selenium in Plots 2 and 3 possibly is affected by erosion-induced OM loss, ineffective land management (terrace systems), Se speciation independent of OM, and disrupted microbial activity essential for Se transformation (Ait Mechedal et al., 2024; Zhang et al., 2023). By 2023, Plot 1 showed a stronger OM– bioavailable selenium relationship (R^2^ = 0.4, *p* < 0.05), while Plots 2 and 3 showed slight increases (R^2^ = 0.04 and 0.07; *p* > 0.05), possibly due to stabilised soil conditions and a shift from deposition to net erosion, enhancing selenium bioavailability (Dinh et al., 2019).

#### Transfer factor of selenium from soil to plants

**Fig. 7:**
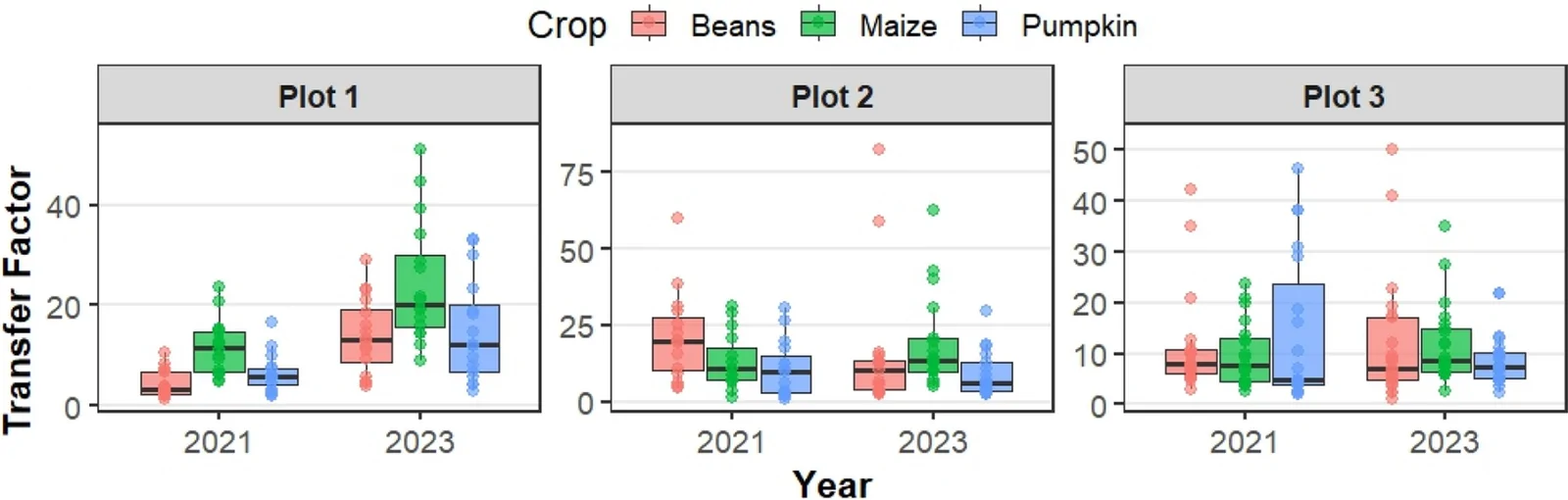
Plant selenium concentration per plot. The global reference selenium concentrations in plants range from 20–50 µg kg^−1^ for maize (WHO/FAO; Lyons et al., 2003; Joy et al., 2015), 30–100 µg kg^−1^ for beans (Broadley et al., 2006), and 10–30 µg kg^−1^ for pumpkin (USDA; Gissel-Nielsen, 1971)

**Fig. 8:**
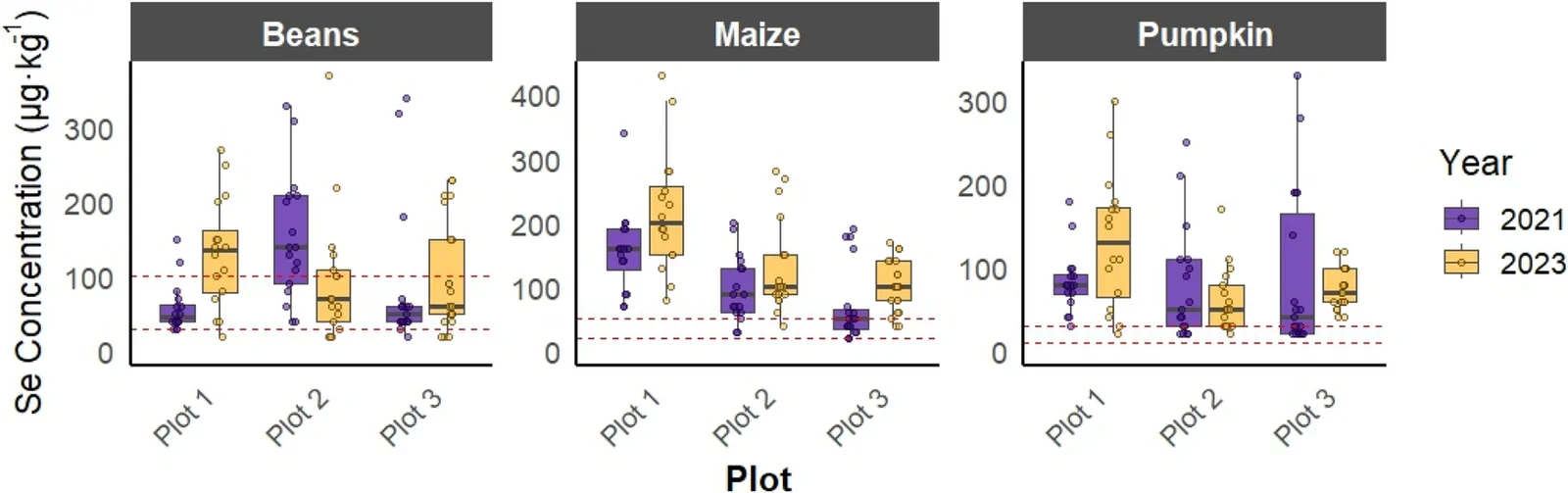
Transfer factors (TF) of selenium across maize, beans, and pumpkin in three experimental plots for the years 2021 and 2023

The transfer factor (TF) results demonstrate how soil erosion and land management practices can affect the transfer of selenium (Se) from soil to crops. TFs ranged from 4 to 24, indicating moderate to high Se bioaccumulation potential across the crops studied (Fig. 8). Maize consistently exhibited the highest TFs, with values in Plot 1 increasing from 11.7 in 2021 to 24.4 in 2023 (+ 12.7). This suggests that in plots where soil fertility was maintained, either through farmers applying fertiliser or through a change in tillage form to reduce erosion and improve nutrient retention, maize benefited most from enhanced Se availability. In contrast, beans exhibited divergent trends across plots, increasing in Plot 1 (+ 9.5%) but decreasing in Plot 2 (–5.4%). These differences likely reflect site-specific soil degradation processes, whereby Plot 2 experienced the highest soil loss, according to Isaboke et al. (2026), while more sustainable management in Plot 1 has preserved bioavailable pools. Pumpkin displayed the most inconsistent uptake, with gains in Plot 1 (+ 8.4) but declines in Plots 2 and 3. This variability underscores pumpkin's sensitivity to soil disturbance, in which erosion stripped topsoil or reduced organic matter, thereby suppressing Se transfer. The high standard deviations, particularly in beans (± 21.6) and maize (± 15.7), highlight strong within-plot variability, which may arise from micro-scale erosion patterns, patchy distribution of organic matter, or differences in soil amendments. The findings suggest that selenium transfer efficiency is not only crop-dependent but also significantly influenced by land use and soil conservation practices. Where erosion is minimised through protective cover, reduced tillage, or organic amendments, selenium uptake improves (Jose et al., 2026). Conversely, erosion-driven nutrient loss diminishes the soil-to-plant pathway, limiting selenium accumulation (Sharma et al., 2015). These results emphasise that sustainable land management is critical not only for soil conservation but also for safeguarding the micronutrient quality of food crops (Figure 7).

#### Influence of soil properties on the transfer of selenium from soil to plants

**Fig. 9:**
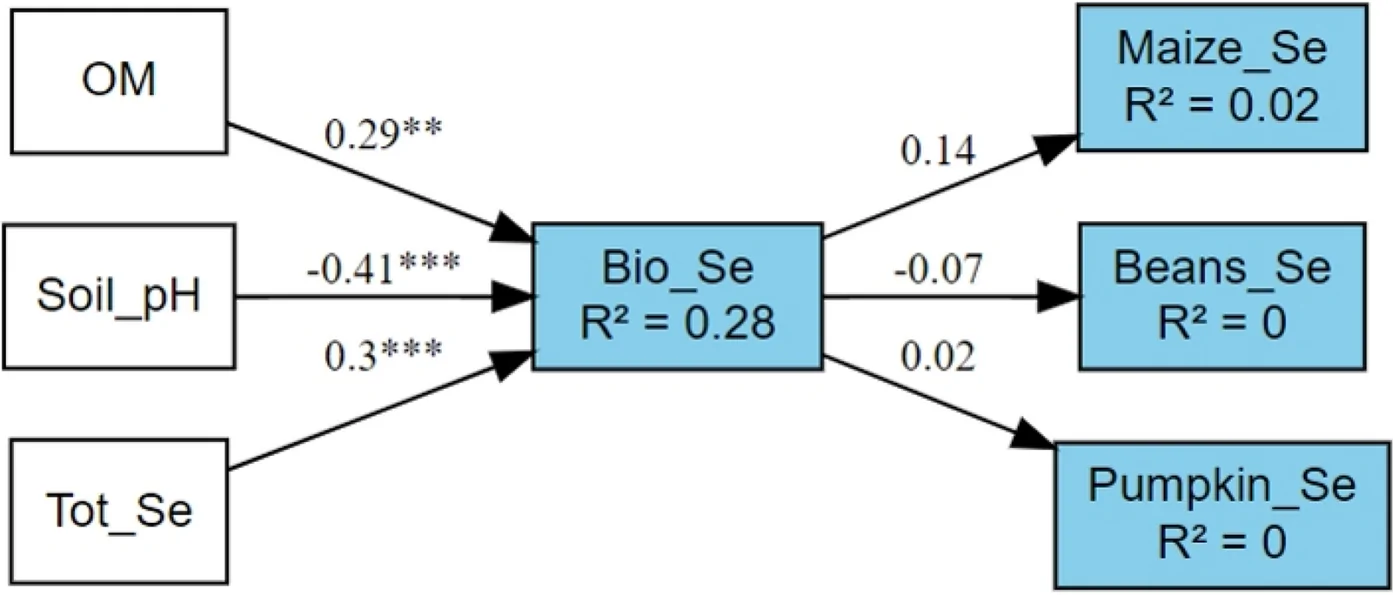
Structural Equation Model (SEM) Path Diagram showing the influence of soil properties (OM, Soil_pH, Total Se (Total selenium)) on bioavailable selenium (Bio_Se) and its effect on crop selenium content (Maize, Beans, Pumpkin). Standardized path coefficients are shown with significance levels (p < 0.05 = *, *p* < 0.01 = **, *p* < 0.001 = ***), and R^2^ indicates explained variance

The structural equation modelling (SEM) analysis examined the relationships among soil properties, OM, soil pH, and total selenium (Total Se) to assess their influence on bioavailable selenium and subsequent uptake in three crop species: maize, beans, and pumpkin. The structural model (Fig. 9) revealed that bioavailable selenium is significantly influenced by Soil_pH (β = − 0.41, *p* < *0.001*), Total Se (β = 0.30, *p* < *0.001*), and OM (β = 0.29, *p* < *0.01*), explaining 28% of the variance in bioavailable selenium (R^2^ = 0.28). However, the subsequent crop-specific selenium uptakes (maize, beans, pumpkin) exhibited very weak relationships with bioavailable selenium, accounting for negligible explained variances: 2% (R^2^ = 0.02) for maize, and 0% for both beans and pumpkin.

Plot level investigation of soil plant selenium transfer revealed, Plot 1, maize, Total Se had a statistically significant positive effect on bioavailable selenium uptake (β = 0.043, *p* < *0.01*), while Soil_pH negatively influenced bioavailable selenium (β = − 0.04, *p* < *0.01*), and OM also had a negative effect (β = − 0.031, *p* < *0.05*) as shown in Fig. 6. Notably, the interaction term between OM and bioavailable selenium (p > 0.05) approached significance, suggesting a possible moderating role of OM. In contrast, Plot 2, beans, showed that only bioavailable selenium had a significant negative effect (β= − 0.025, *p* < 0.05), while the other predictors were not significant. Plot 3 (pumpkin) demonstrated no significant effects from any variable, suggesting a decoupling between soil selenium dynamics and selenium uptake in this crop.

Although maize showed the clearest temporal response among the crops studied, its Se accumulation was only weakly explained by soil bioavailable Se. Soil properties, particularly soil pH, total Se, and OM, significantly influenced bioavailable Se. Soil pH had a significant negative effect on bioavailable Se (β = −0.41, *p* < 0.001), while total Se (β = 0.30, *p* < 0.001) and OM (β = 0.29, *p* < 0.01) had significant positive effects, collectively explaining 28% of the variation in bioavailable Se (R^2^ = 0.28). However, the subsequent transfer of Se into plant tissues was weak and highly crop-specific, with bioavailable Se explaining only 2% of the variation in maize Se accumulation (R^2^ = 0.02) and none of the variation in beans and pumpkin (R^2^ = 0). These weak relationships indicate that soil bioavailable Se alone is insufficient to explain Se accumulation in crops and suggest that physiological and species-specific characteristics, as well as other soil–plant processes, may contribute to Se uptake and assimilation (Chen et al., 2026).

Selenium accumulation also varied among crops and between sampling periods, indicating that plant responses were influenced by both crop type and site conditions. Maize showed a significant increase in Se concentration in Plot 1 (*p* < 0.05), whereas changes in Plot 2 and Plot 3 were not significant (*p* > 0.05). Beans showed a highly significant increase in Plot 1 (*p* < 0.01), but changes in Plot 2 and Plot 3 were not significant (*p* > 0.05). Pumpkin similarly showed a significant increase only in Plot 1 (*p* < 0.05), with no significant changes in Plot 2 or Plot 3 (*p* > 0.05). These crop-specific and spatially variable responses further support the interpretation that Se accumulation in plant tissues is not influenced by soil bioavailable Se alone (Guarino et al., 2022). Although maize showed the clearest temporal response among the crops studied, its uptake should not be interpreted as being governed primarily by soil bioavailable Se. Results suggest that improving soil Se availability through management may support the soil Se pool, but may not necessarily result in proportional increases in crop Se concentrations (Rosinger et al., 2025). Targeted agronomic interventions, including selection of crop varieties with greater Se uptake efficiency and appropriate Se biofortification approaches, may therefore be required where increasing crop Se concentration is an objective.

## Conclusion

This study indicates that soil erosion and land-use management are associated with Se bioavailability and redistribution in agricultural soils of Nandi County, Kenya. Soil pH (β = −0.41, *p* < 0.001), organic matter (β = 0.29, *p* < 0.01), and total Se (β = 0.30, *p* < 0.001) collectively explained 28% of the variation in bioavailable Se. However, bioavailable Se explained only 2% of the variation in maize Se accumulation (R^2^ = 0.02) and negligible variation in beans and pumpkin (R^2^ = 0), suggesting that soil Se availability alone may not adequately explain Se accumulation in crops. The observed differences may also reflect crop-specific and physiological factors involved in Se uptake and assimilation. Temporal and spatial patterns indicated differences in Se availability among plots with contrasting land-use and erosion conditions. In Plot 1, net erosion (− 0.58 t ha⁻^1^ yr⁻^1^) was accompanied by a 35% decline in bioavailable Se between 2021 and 2023, whereas Plot 3 showed a 42% increase despite a shift from deposition (+ 0.72 t ha⁻^1^ yr⁻^1^) to erosion (− 0.43 t ha⁻^1^ yr⁻^1^). Plot 2, which experienced persistent soil loss, maintained relatively low Se availability. These patterns suggest that erosion and changes in land use may contribute to variation in soil Se pools, although other soil processes may also be involved.

Crop responses varied among plots and sampling periods, with maize reaching the highest Se concentration (0.187 mg kg⁻^1^ in Plot 1), while beans and pumpkin showed less consistent accumulation. Although several crop Se concentrations were low relative to the reference ranges considered in this study, these findings may indicate a potential concern regarding Se adequacy rather than evidence of dietary Se deficiency. The findings suggest that soil management and erosion control may contribute to maintaining soil Se availability, but increases in bioavailable Se may not necessarily result in proportional increases in crop Se concentrations. Where improvement of crop Se concentration is desired, soil conservation could be considered alongside targeted agronomic approaches, including Se fertilisation, foliar application, crop diversification, and selection of varieties with greater Se accumulation efficiency.

## Supplementary Information

Below is the link to the electronic supplementary material.

Supplementary file1 (XLSX 1175 KB)

## Data Availability

All data supporting the findings of this study are available within the paper and its Supplementary Information.
